# Child health nursing consultation and competencies for Advanced Practice Nurses*

**DOI:** 10.1590/1980-220X-REEUSP-2023-0269en

**Published:** 2024-05-27

**Authors:** Keila Gisele Lima Reis, Nayara Vilela de Farias Serranegra, Andrea Liliana Vesga Varela, Patricia Aline de Almeida, Marilia Orlandelli Carrer, Carla Pereira Barreto, Jessica Kelly Ramos Cordeiro, Claudia Santos Martiniano, Manoel Vieira de Miranda, Daiana Bonfim

**Affiliations:** 1 Hospital Israelita Albert Einstein, Faculdade Israelita de Ciências da Saúde Albert Einstein, São Paulo, SP, Brazil.; 2 Hospital Israelita Albert Einstein, São Paulo, SP, Brazil.; 3 Universidade Estadual da Paraíba, Departamento de Enfermagem, Campina Grande, PB, Brazil.

**Keywords:** Professional Competence, Advanced Nursing Practice, Pediatric Nursing, Primary Health Care, Competencia Profesional, Enfermería de Práctica Avanzada, Enfermería Pediátrica, Atención Primaria de Salud, Competência Profissional, Prática Avançada de Enfermagem, Enfermagem Pediátrica, Atenção Primária à Saúde

## Abstract

**Objective:**

To analyze nurses’ practice in child health nursing consultations and the presence of care management competencies proposed for Advanced Practice Nurses (APN).

**Method:**

Multicenter, exploratory sequential mixed methods research, carried out in 17 Basic Health Units in four Brazilian cities. Collection was carried out from May to July 2022 through filming of consultation and analysis of medical records. Consultations with compliance with the Nursing Process ≥50% were analyzed to identify the competencies proposed for APN.

**Results:**

24 child consultations carried out by 12 nurses were filmed. In the quantitative analysis, 11 nursing consultations, carried out by seven nurses, achieved ≥50% Nursing Process compliance. In the qualitative analysis of these consultations, some APN competencies in care management were identified, but incomplete.

**Conclusion:**

child health nursing consultations present weaknesses in carrying out the Nursing Process, and nurses demonstrated a partial and superficial application of the care management competencies proposed for APN.

## RESUMO

**Objetivo:**

Analisar a prática de enfermeiros nas consultas de enfermagem em saúde da criança e a presença das competências de gestão do cuidado propostas para o Enfermeiro de Prática Avançada (EPA).

**Método:**

Estudo multicêntrico, método misto sequencial exploratório, realizado em 17 Unidades Básicas de Saúde em quatro cidades brasileiras. A coleta foi realizada de maio a julho de 2022 através de filmagem da consulta e análise dos registros em prontuário. As consultas com cumprimento do Processo de Enfermagem ≥50% foram analisadas para identificar as competências propostas para EPA.

**Resultados:**

Foram filmadas 24 consultas de crianças realizadas por 12 enfermeiros. Na análise quantitativa, 11 consultas de enfermagem, realizadas por sete enfermeiros, alcançaram cumprimento ≥50% Processo de Enfermagem. Na análise qualitativa dessas consultas, algumas competências do EPA em gestão do cuidado foram identificadas, porém incompletas.

**Conclusão:**

As consultas de enfermagem em saúde da criança apresentam fragilidades na realização do Processo de Enfermagem, e os enfermeiros demonstraram uma aplicação parcial e superficial das competências de gestão do cuidado propostas para o EPA.

## RESUMEN

**Objetivo:**

Analizar la práctica del enfermero en consultas de enfermería en salud infantil y la presencia de habilidades de gestión del cuidado propuestas para el Enfermero de Práctica Avanzada (EPA).

**Método:**

Estudio multicéntrico, método mixto secuencial exploratorio, realizado en 17 Unidades Básicas de Salud de cuatro ciudades brasileñas. La recolección se realizó de mayo a julio de 2022 mediante filmación de la consulta y análisis de historias clínicas. Se analizaron las consultas con cumplimiento ≥50% del Proceso de Enfermería para identificar las competencias propuestas para EPA.

**Resultados:**

Se filmaron 24 consultas infantiles realizadas por 12 enfermeras. En el análisis cuantitativo, 11 consultas de enfermería, realizadas por siete enfermeros, alcanzaron ≥50% de cumplimiento del Proceso de Enfermería. En el análisis cualitativo de estas consultas se identificaron algunas competencias del EPA en la gestión del cuidado, pero incompletas.

**Conclusión:**

Las consultas de enfermería en salud infantil presentan debilidades en la realización del Proceso de Enfermería, y los enfermeros demostraron una aplicación parcial y superficial de las habilidades de gestión del cuidado propuestas para el EPA.

## INTRODUCTION

Primary Health Care (PHC) is a powerful scenario for the development of Advanced Practice Nursing, especially in health care of priority groups, historically incorporated into nursing care, such as children. It is understood, therefore, that Advanced Practice Nursing “refers to accurate and expanded health interventions provided by nurses who, with advanced capabilities, influence clinical health outcomes and provide direct health services to individuals, families and communities”(ICN, 2020, p. 9)^(1)^.

In this regard, nursing consultation (NC) is a potential practice, as it is a private activity, supported by technical-scientific knowledge, identifying health-illness situations and providing qualified and safe care to users, in which advanced nursing interventions may be present, despite the fact that consultation is not the only space for the development of advanced practices, nor is it an advanced practice itself^(2)^. However, further progress is still needed in nurses’ autonomy and clinical practice so that access to care can be expanded in its resolution in different regions of Brazil^(2)^.

Internationally, and more recently in Brazil, discussions and incentives for nurses’ work in PHC, in a decisive and expanded way, have gained ground, with incentives from the Pan American Health Organization (OPAS) and the Federal Nursing Council (COFEN – *Conselho Federal de Enfermagem*) to Advanced Practice Nurse (APN) training^(3)^. The International Council of Nurses (ICN) defines APN as:

“[…] a registered nurse who has acquired the expert knowledge base, complex decision-making competencies and clinical competencies for expanded practice, the characteristics of which are shaped by the context and/or country in which she/he is credentialed to practice. A master’s degree is recommended for entry level”(ICN, 2020, p. 9)^(1)^.

In Brazil, we still do not have training and regulation for APN, but there are movements in its favor, such as the COFEN Technical Note 001/2023 on Advanced Nursing Practice in Brazil: context, concepts, actions undertaken, implementation and regulation, and initiatives in the area of child health, with Brazilian studies that discuss expanding the scope of advanced nursing practices in hospitals and outpatient clinics^(4,5,6)^. Furthermore, in PHC, there are discussions about the Integrated Management of Childhood Illness Illnesses (IMCI) strategy implementation, officially adopted by the Brazilian Ministry of Health in 1996, as an initiative to expand nurses’ practices for PHC, based on three basic pillars (human resource training, health service reorganization, and health, family and community education). These pillars sought to identify and manage illnesses in children under 5 years old with integrated care behaviors, describing how professionals should assess and classify sick children aged 2 months to 5 years old, treat children, advise mother/father or responsible, provide care to children from 1 week to 2 months of age and carry out a follow-up consultation^(7)^.

Thus, this research seeks to answer the following questions: how is NC carried out in child health in PHC? Are care management competencies proposed for APN present in nurses who perform NC on children in PHC?

This study, therefore, aimed to analyze nurses’ practice in NC in child health as well as the presence of care management competencies proposed for APN.

## METHOD

### Study Design

**Figure 1:**
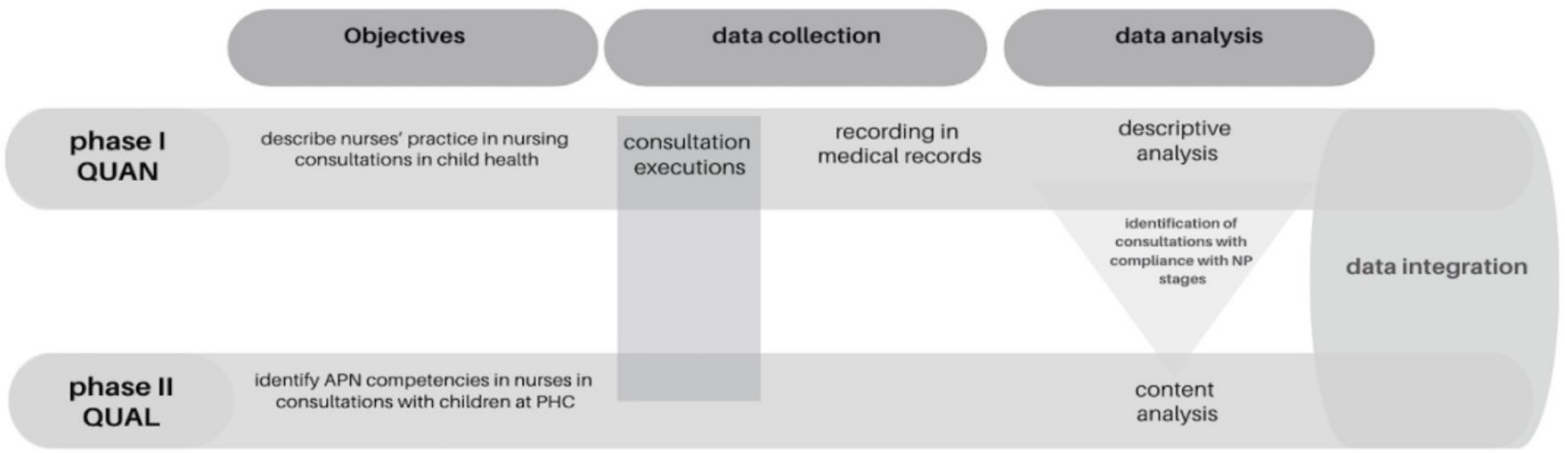
Representative diagram of the study design.

This is multicenter, exploratory, mixed methos research, with an explanatory sequential design^(8)^. The phases of the study (quantitative and qualitative) are represented in Figure 1.

### Place and Period

Data were collected between May and July 2022 in 17 Basic Health Units (BHU) in four Brazilian municipalities: São Paulo, SP, Manaus, AM, Carneiros, AL, and Parelhas, RN.

### Population

Nurses who worked at the BHU in full exercise of their duties on the day of data collection and children up to 12 years 11 months and 29 days attended were included, whose parents/guardians agreed to participate in the research. Nurse managers and children receiving emergency care were excluded.

### Data Collect

Data were collected during execution and registration in NC records. Previously, a pilot test was carried out to adjust the collection process for carrying out consultation, presenting the research to BHU nurses, verifying consents and planning collection in each municipality.

Consultation execution was recorded through filming and recording of consultations, with direct and non-participatory observation. For this, the presence of two researchers (A and B) was necessary. Researcher A approached and invited children and their guardians while they were waiting for care at the BHU. Researcher B positioned the two recording cameras, model GoPro^®^ Hero 9, with one camera fixed to the nurses’ body to capture inter-consultations or case discussions outside the office, and the other was fixed in the office to capture the sound and image of the consultation. The cameras were turned on by researcher B, who presented each user’s identification code, left the room, and, after consultation was over, entered and turned off the cameras, ensuring data confidentiality and safe storage on an external hard drive and institutional cloud software.

To capture data from filmed consultations, in the quantitative phase, a checklist (REDCap) was created containing essential elements for carrying out NC in child health based on the stages of the Nursing Process (NP)^(9)^, Primary Care Record 33^(10)^ and Child Health Record^(11)^. Furthermore, recording in medical record was also captured using a checklist (REDCap), covering the NP stages adapted for records in the SOAP format^(12)^.

To capture APN competencies in filming, the competencies proposed by Cassiani et al.^(13)^ were used, consisting of seven domains. In the present study, for NC extraction purposes, only the care management domain was considered, consisting of three themes, namely: Focus on care (three competencies); Assessment and diagnosis (seven competencies); and Provision of care (ten competencies).

### Data Analysis

For quantitative analysis, descriptive statistics were performed. For qualitative analysis, content analysis was used^(14)^. Data integration was carried out by connecting quantitative and qualitative results.

### Ethical Aspects

The research was approved by the Research Ethics Committee (Opinion 5,362,332) of *Hospital Israelita Albert Einstein*, São Paulo. In accordance with Resolution 466/12^(15)^, the Informed Consent Term (ICF), the Informed Assent Form (IAF), image and voice sound authorization (filming) were applied for nurses, children and their legal guardians.

## RESULTS

After applying the inclusion criteria, 24 consultations for children up to 12 years 11 months and 29 days were selected, with a mean age of 3 years, distributed in the municipalities of São Paulo (41.3%), Parelhas (37.5%), Manaus (16.7%) and Carneiros (4.2%). The participating children are mostly male (58%), white (54.2%), with a family income between one and two minimum wages (70.8%), living in urban areas (88%), who seek out the BHU mostly for childcare (58%) and followed by acute events (33.3%).

Consultations were carried out by 12 nurses, mostly female (91.7%), with an exclusive use office (83.3%), using electronic medical records in the unit (66.7%). Half of nurses have experience in their profession between six and ten years, and the other half, more than ten years. All nurses reported having a postgraduate degree, but the majority in other areas (61.5%).

In relation to the courses taken by nurses in the last year, which could be one or more, 46.2% of nurses responded that they had taken a course in child health and 23.1% in NP. Most nurses use ministerial protocols (69.2%), followed by the basic care record (61.5%). However, more than half (58.3%) reported difficulties in performing NC, and only 25% use a standardized instrument for NP.

NC in child health, analyzed through the stages of NP, at the time of execution, are described in Table 1.

**Table 1:** Stages of the Nursing Process analyzed during the execution of nursing consultations on child health in Primary Health Care – São Paulo, SP, Brazil, 2022.

Nursing Process stages	No n(%)	Yes n(%)
**1. Nursing history**		
Did the nurse request the Child Health Record at any point during consultation?	8(33)	16(67)
Did the nurse perform an assessment of the child’s growth?	12(50)	12(50)
Did the nurse assess the child’s development?	14(58)	10(42)
Did the nurse assess the child’s emotional development?	16(67)	8(33)
Did the nurse assess the child’s vaccination status?	8(33)	16(67)
Did the nurse assess aspects of the child’s nutrition?	5(21)	19(79)
Did the nurse assess the child’s fundamental rights (education, daycare, benefits)?	16(67)	8(33)
Did the nurse assess supplementation (vitamin A/iron/vitamin D)? (applied to children under 5 years old)	8(50)	8(50)
Did the nurse perform a specific physical examination for the reason for consultation?	12(50)	12(50)
Did the nurse weigh, measure and measure the head circumference (applied to children under 2 years old)?	0(0)	15(100)
Did the nurse perform the visual acuity test? (applied to people over 5 years old)	9(100)	0(0)
**2. Nursing Diagnosis**		
Did the nurse tell the person in charge a Nursing Diagnosis?	24(100)	0(0)
**3. Planning**		
Did the nurse agree on the care plan?	13(54)	11(46)
Did the nurse write a nursing prescription?	11(46)	13(54)
**4**. **Implementation**		
Did the nurse apply intervention elements in relation to the first reason for consultation?	10(42)	14(58)
Did the nurse provide guidance on accident prevention?	21(88)	3(12)
Did the nurse provide guidance on healthy eating (breastfeeding, included if applicable)?	11(46)	13(54)
Did the nurse address the child’s rights?	20(83)	4(17)
Did the nurse talk about vaccination?	10(42)	14(58)
Did the nurse address elements of basic care (body hygiene)?	18(75)	6(25)
Did the nurse discuss the importance of playing in childhood?	23(95.8)	1(4.2)
Did the nurse address oral care issues?	19(79)	5(21)
Did the nurse address the aforementioned complaint?	4(17)	20(83)
**5. Assessment**		
Did the nurse mention the need for a new meeting?	3(12)	21(88)
Did the nurse focus on the complaint, but scheduled the child’s regular care with the user for another meeting?	13(54)	11(46)

Analysis of clinical communication practices during NC in child health revealed that the majority of nurses greeted and identified the person (88%) and there was attention to comfort and privacy during the interaction (95.8%). Using open-ended questions at the beginning of the interview was positive (91.7%), as was the encouragement to continue the report and verbalize feelings and concerns (67%). However, most nurses (62%) did not introduce themselves during consultation. The practice of synthesizing information and involving the person in planning was observed in a significant portion of consultations (67%), but formal closure of consultation was less frequent (42%).

Consultations were recorded % in electronic medical records (83%). Considering the stages of the NP, nursing history was partially recorded with the presence of subjective data in 50% of consultations, 80% of objective data, 65% of assessment, and physical examination was recorded in only 55%.

Regarding Nursing Diagnosis (ND), there is little record, being present in only 20% of consultations, and, of this total, 15% had ND related to nursing history. However, the use of the International Classification of Primary Care (ICPC) was found in 70% of these, but only 45% of ICPC records were related to nursing history. In the planning stage, nursing prescription was partially observed in a little more than half of consultations (55%). In the implementation stage, only 10% of consultations are recorded and only partially. During assessment, the plan was revised in 20% of consultations.

**Figure 2:**
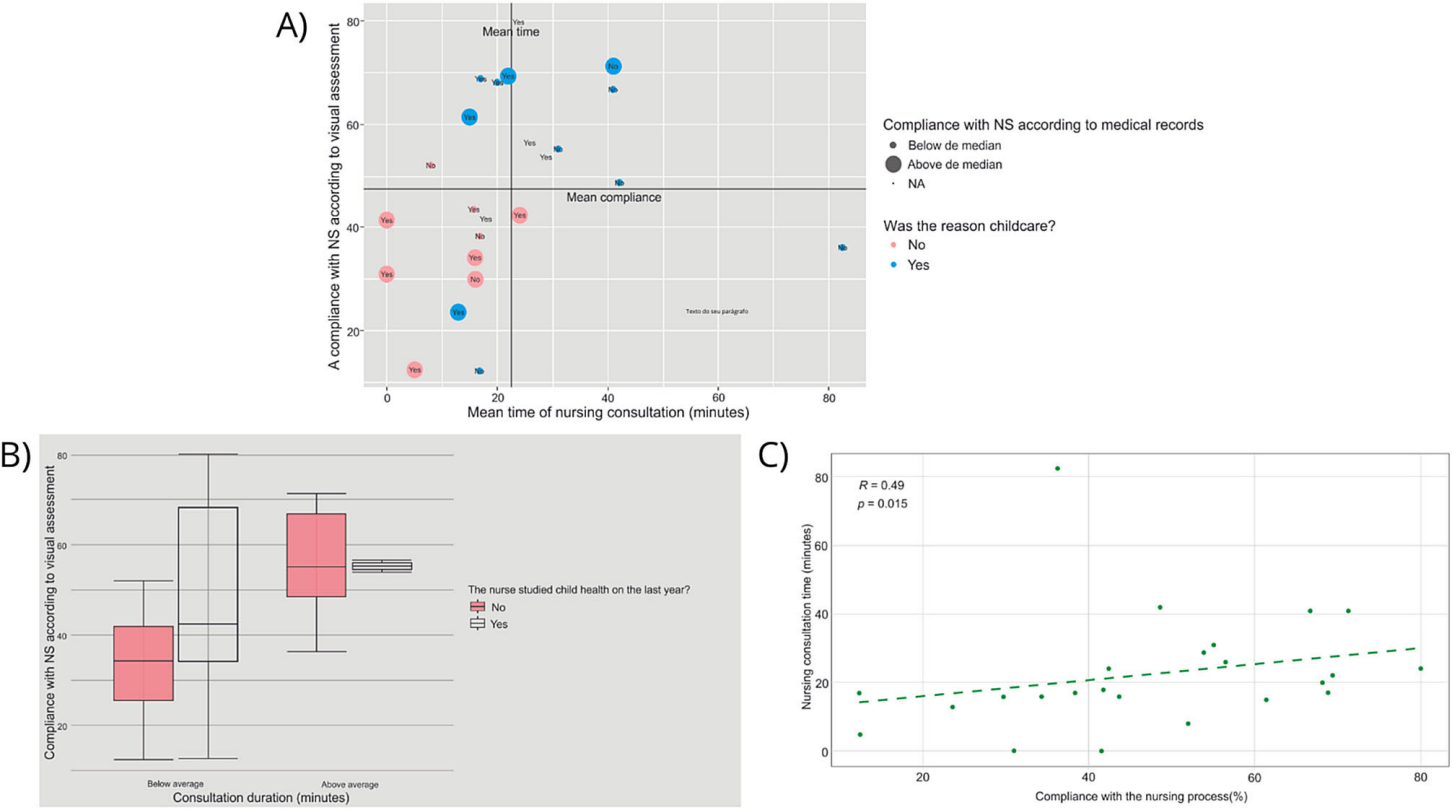
Relationship between the reason for consultation, course taken in the last year, consultation time and compliance with the Nursing Process stages.

When analyzing the relationship between the reason for consultation and the percentage of compliance with the NP stages, it was observed that children whose reason for consultation was childcare had a mean compliance with NP greater than or equal to 50% (Figure 2.A). Furthermore, it is possible to observe a relationship between nurses who took the child health course in the last year with greater compliance with the NP stages (Figure 2.B). There is also an association (Figure 2.C) between time spent in consultations and compliance with the nursing stages in the videos (p-value = 0.015).

**Figure 3:**
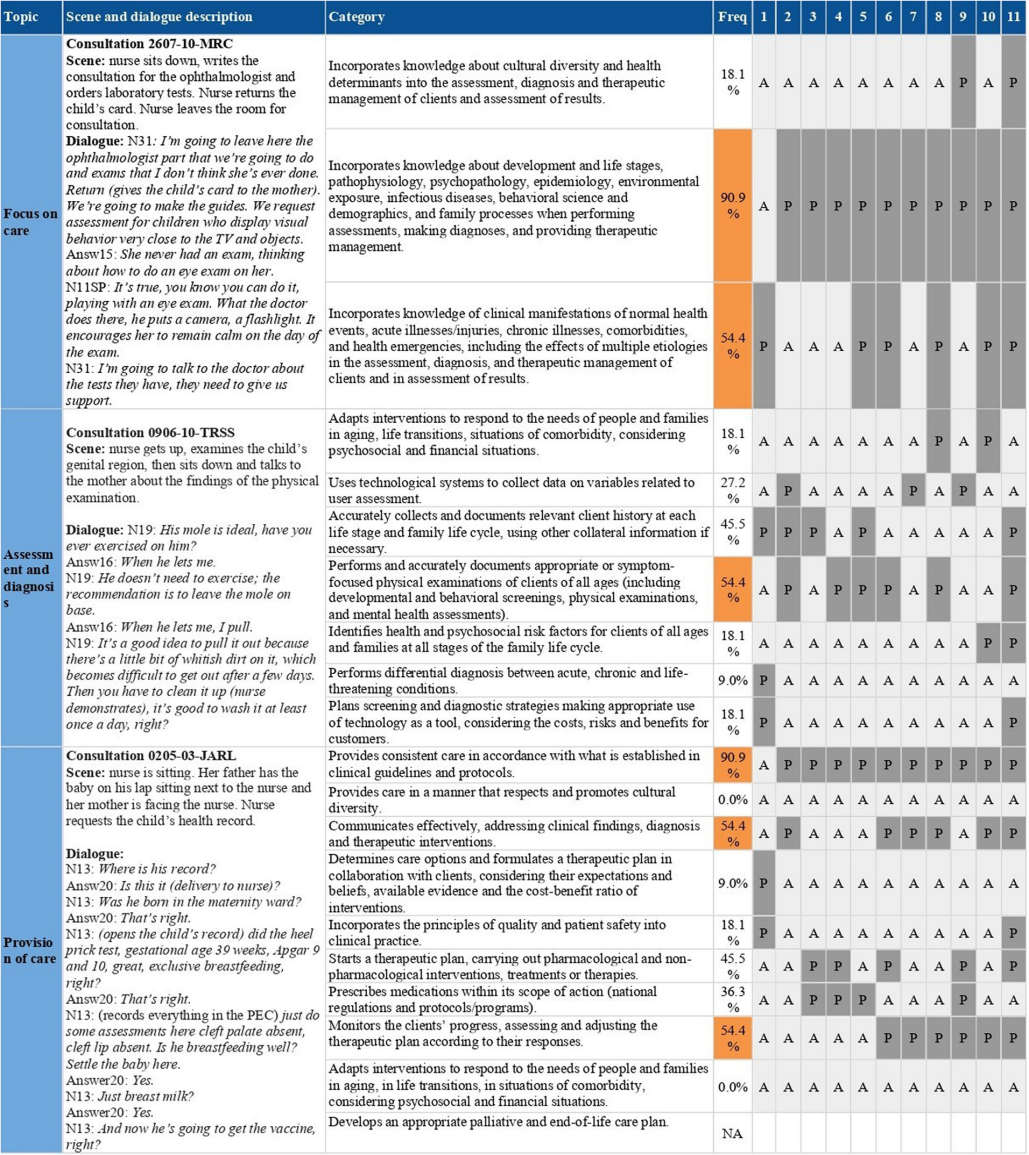
Competencies proposed for Advanced Practice Nurses in Primary Health Care in care management, assessed in child health consultations.

Of the total number of consultations analyzed, 11 reached ≥50% of the NP and were selected for analysis of the care management competencies proposed for APN^(13)^, as shown in Figure 3.

The 11 consultations analyzed for APN competencies, distributed in Parelhas (54.4%), São Paulo (36.3%) and Manaus (9%), were carried out by seven nurses, of whom 71.4% took a child health course in the last year and 28.5% in NP. Thus, 85.8% have an exclusive use office; 85.8% use the Basic Care record; and 57.1% reported having difficulty performing NC. Furthermore, they were performed on children with a mean age of 1.3 years, and the majority (81.8%) referred to childcare consultation as the main reason, and in 18.1% of these consultations, inter-consultation with a medical professional was observed.

### Data Integration

The results were integrated from the NP analysis of consultations with the recognition of care management competencies, proposed for APN in PHC. In both approaches, the NC’s fragility was partially highlighted.

The NP stage regarding nursing history was elementary, especially in growth and development assessment. Likewise, in the analysis of APN competencies, nurses superficially incorporated the competency of accurately collecting and documenting children’s relevant history at each stage of life and the family life cycle, using other collateral information. The ND was absent in execution, infrequent in the medical record, and did not advance when analyzed in the field of Advanced Practice Nursing, such as carrying out the differential diagnosis between acute, chronic and life-threatening conditions.

Regarding the planning stage, nursing prescription presented itself positively, an aspect also found regarding the competency in prescribing medications within its scope of professional activity proposed for APN, even if partially, considering the nursing protocols.

For the implementation stage, nurses superficially addressed the essential elements in childcare consultation, such as healthy eating, including breastfeeding, vaccination, growth, development, oral health, body hygiene, among others. Likewise, partially, when analyzing the competencies for APN, the competency of cultural diversity and health determinants in the provision of care was barely present, respecting children’s cultural diversity and determining therapeutic care options in collaboration with children and/or their guardians.

Regarding the assessment stage, a nurse scheduled a new meeting for the majority of consultations carried out. Considering that the study population consisted of children with a median age of one year, longitudinal monitoring is expected, strongly implemented nationally, a fact recognized by the high frequency (90.9%), with the incorporation of competency to provide consistent care in accordance with what is established in clinical guides being identified, but partially and superficially.

Thus, in general, NC in child health presents weak points in relation to the NP and little recognition (35.1%) of the presence, i.e., partially, of the competencies proposed for APN.

## DISCUSSION

With the analysis of NC on child health in PHC, weaknesses in their execution and registration were evident. In this case, the competency profile for care management proposed for APN in PHC was confirmed, but in an incipient, fragile and partial manner, especially the assessment and diagnosis domain.

Studies revealed that nurses’ practice, based on NP stages, improves child care safety and favors comprehensive and longitudinal care. However, it is still little incorporated by most nurses, a fact also confirmed in the study carried out in the east of the state of São Paulo with nurses in childcare consultations working in the Family Health Strategy, in which professionals, object of study in the research, reported that structural and personal difficulties and the influence of beliefs, values and social conditions of the assisted population interfere with child care^(16)^.

In relation to the NP stages, the moment in which nurses prescribed medications recommended by national programs, such as ferrous sulfate, vitamin A and vitamin D, deserves a positive highlight. A result also found in the study “Nursing Practices in the Context of Primary Health Care: National Mixed Methods Research” (*Práticas de Enfermagem no Contexto da Atenção Primária à Saúde: Estudo Nacional de Método Misto*) identifies which medications nurses can prescribe. Most nurses stated that they prescribe ferrous sulfate and other supplements^(17)^.

However, when referring to care plan, it is observed that nurses still rarely agree and implement essential care for child health, different from that found in a systematic review, in which nurses’ clinical competency was statistically significant in explaining the positive relationship between parents’ adherence to care plan^(18)^.

Another aspect is the relationship between NC duration and the association with compliance with nursing stages, which, despite being present in this study, is still an aspect that requires further investigation. Mixed methods research had a mean video-recorded consultation duration of 10.97 minutes (± 4.13), showing, for instance, that the way consultations are conducted can be more important than their duration^(19)^.

Internationally, nurses practice is discussed mainly due to educational training and the development of professional competencies. In developed countries, such as Canada, United States, United Kingdom, New Zealand, Australia, APN roles are regulated, and nurses can work autonomously and collaboratively in PHC for the adult and child population^(18)^.

In this context of expanding practices, it is important to highlight that, in developing countries, there were positive movements carried out by the IMCI strategy that expand the scope of nurses’ work in child health care, whose objective is to identify signs of danger^(7)^. However, once the IMCI strategy is implemented, its monitoring must be carried out routinely, in order to identify the main difficulties faced by professionals. A study in Ethiopia demonstrated that the most common problems encountered in IMCI implementation are related to lack of training, medications, essential supplies and especially supervision and follow-up visits^(20)^. Another quantitative study, carried out in Colombia, revealed that the assistance provided to children under five years of age remains incomplete, as it does not provide the minimum necessary for adequate implementation of IMCI in the country^(21)^.

However, an assessment carried out among five countries, including Brazil, in which a survey was carried out in 24 health units in four states in the Northeast region, revealed that nurses trained in IMCI showed good performance when compared to other professionals^(22)^, but, despite its relevance, it is a strategy that is still little present in professional practice.

In the Center-West, a study showed that among the reasons for not using IMCI are the lack of training and lack of knowledge of the strategy by professionals^(23)^. In 2023, there are few reports by PHC nurses who say they use the IMCI strategy, in addition to specific protocols and guidelines^(17)^.

Currently, there are other strategies underway to expand the scope of nurses’ practice in PHC, such as the award for the nursing innovation laboratory, an initiative created by PAHO/WHO and COFEN. This initiative presents the implementation of clinical nursing protocols in the city of Florianópolis, SC, including child health, expanding access to services offered by the Brazilian Health System and with the core of facilitating the identification of signs of the severity of prevalent diseases, but without losing focus on monitoring children’s healthy growth and development^(24)^.

However, despite advances in nursing practice, with Advanced Practice Nursing, internationally, and expansions of scope in Brazil, the present study shows that nurses have made little progress in competencies involving diagnosis, screening, therapeutic plan, cultural diversity and consistent social determinants. In relation to development and life stages, they provide consistent care in accordance with what is established in clinical guides and protocols, however with limitations, as it is possible to observe fragile clinical reasoning and the performance of NC guided mainly by the Child Health Record, not advancing towards identifying and addressing children’s and family’s needs. This fact is also evidenced by a study carried out in the Brazilian Center-West, which identifies aspects such as child growth, being carried out using the curves from the Child Health Record, but development being assessed partially in most consultations^(25)^.

Therefore, in order to move forward with Advanced Practice Nursing implementation and training in Brazil, addressing child health in PHC, it is important to consider the health model in force in Brazil. Despite advances in child health, we still have weaknesses, as the health model is still centered on the biomedical model and prevention and promotion actions are little valued. Additionally, the existence of different PHC models, such as Manaus, which has a specialized service called Comprehensive Child Care Center (CAIC – *Centro de Atenção Integral à Criança*)^(26)^ managed by the state, displaces care coordination and gateway from PHC.

In this context, APN emerge, professionals trained to meet child health demands aiming at centered care, taking into account social and cultural determinants, which can be formed according to the Brazilian health system’s needs, considering the current scenario of infant mortality and the role of nurses in PHC. However, jointly and concomitantly, there is a need to invest in continuing education opportunities for generalist nurses who work in PHC, seeking to develop competencies, especially in topics such as NP with an emphasis on essential care for child health. Finally, there is an urgent need for a joint debate on expanding the scope of practices with APN and nurse qualifications.

The limitations of this study are related to the possible change in behavior expected in the methodological process of filming the consultation, which can generate shyness and embarrassment for both nurses and users. Additionally, sample size and selection may underestimate measurements due to selection and classification bias. However, this investigation presents powerful results to support the discussion on this topic, involving several agents, such as the institutions responsible for training nurses, professional bodies, local management and, mainly, nurses working in PHC.

## CONCLUSION

The study showed that, to strengthen quality nursing care in PHC, it is necessary to jointly advance discussions and proposals to expand the scope of practices with APN for the qualification of nurses who work in PHC, as there is weakness in NC execution and registration through the NP, especially in the assessment and diagnosis stages, as well as when analyzing the competencies in the care management domain proposed for APN, which are still incipient.

To overcome this weakness, it is necessary to expand the incorporation of continuing education actions as well as a strong curricularization of the NP applied to nurses’ clinical practice in PHC. Furthermore, the strengthening of *lato sensu* graduate programs, along the lines of residency, provides nurses with a strong clinical base, in addition to professional master’s degrees focused on the implementation of evidence-based practices, structuring contributions to APN training in PHC.
